# Pore architecture governs tannic acid release from porous *Poria cocos* polysaccharide beads for headspace antioxidant preservation of fruit

**DOI:** 10.1016/j.fochx.2026.104670

**Published:** 2026-10-22

**Authors:** Xichen Qiao, Jinwang Zou, You Zhang, Chang Zhou, Ruyan Qian, Xizun Tan, Shipeng Wu, Changchun Li, Yao Wu, Hairong Xiong, Zongbao Zhou

**Affiliations:** a Hubei Key Laboratory of Resource Utilization and Quality Control of Characteristic Crops, College of Life Science and Technology, Hubei Engineering University, Xiaogan 432000, China; b Department of Stomatology, Shanghai Eighth People's Hospital, Jiangsu University, Shanghai 200235, China

**Keywords:** *Poria cocos* polysaccharide, Tannic acid, Porous hydrogel beads, Active packaging, Fruit preservation

## Abstract

Postharvest fungal decay and water loss waste substantial fruit, while sulfur dioxide pads leave sulfite residues and perform unevenly across commodities. Herein, tannic acid (TA) was loaded into *Poria cocos* polysaccharide (PCP) hydrogel beads via hydrogen-bond assembly; salt porogen leaching with gradient annealing created interconnected, PCP-tuned pore channels. Pore sizes of 24.9–36.4 μm shifted release from Fickian to anomalous transport, and medium-porosity PTB-2 was selected for balanced release, high moisture sorption, and bead quality. PTB-2 achieved 97.4% cumulative TA release (*n* = 0.55, R^2^ = 0.998), and with headspace DPPH scavenging reaching 66.9% at 48 h, linearly correlated with released TA (R^2^ = 0.982). Over 14 days, PTB-2 reduced grape decay to 19.2% (vs 76.7% control; UVASYS 14.2%) and cherry tomato decay to 21.3% (vs 58.6% control; UVASYS 53.1%), retaining anthocyanin and lycopene. This pore-tuned polyphenol platform offers a natural, broad-spectrum route to residue-minimized active fruit packaging.

## Highlights

• Annealed *Poria cocos* polysaccharide/tannic acid hydrogel beads (PTB) were prepared.
• Pore architecture regulated tannic acid release from Fickian to anomalous transport.
• PTB-2 matched sulfur dioxide pads on grapes, outperformed on tomatoes.
• PTB-2 showed no detectable acute oral toxicity in a 28-day mouse study.

## 1 Introduction

Postharvest losses of fresh fruits and vegetables account for up to 40% of total production worldwide, with fungal infection and transpiration-driven water loss identified as the leading causes of quality deterioration (Liu et al., 2025; Shankar et al., 2024). Cold storage and synthetic fungicides remain the predominant preservation strategies. Refrigeration is effective but energy-intensive and inaccessible in many postharvest supply chains of developing regions (Stathers et al., 2020), whereas chemical fungicides such as SO₂-generating pads and thiabendazole raise persistent concerns over chemical residues, pathogen resistance, and consumer acceptance, particularly in the context of growing demand for clean-label and minimally processed foods (Fernandes et al., 2024). In this context, active packaging systems that continuously release natural antimicrobial or antioxidant agents into the package headspace have emerged as a non-contact, residue-minimizing strategy capable of bridging the gap between preservation efficacy and consumer safety.

Polysaccharide-based hydrogels, including those derived from chitosan, alginate, cellulose, and starch, are particularly attractive carriers for active packaging because of their biodegradability, biocompatibility, high moisture uptake, and tunable network architecture (Ali et al., 2025). Tannic acid (TA), a hydrolyzable plant polyphenol with generally recognized as safe (GRAS) status, carries multiple galloyl groups whose phenolic hydroxyls donate hydrogen atoms to quench radicals and interact with bacterial membrane proteins and lipids, underpinning its broad-spectrum antimicrobial and radical-scavenging activities at relatively low concentrations. TA has been incorporated into pectin/gelatin and alginate matrices for fruit preservation (Li et al., 2025; Zhang et al., 2024). Wang et al. (2025) further showed that TA-functionalized polysaccharide coatings delayed anthracnose and prolonged the shelf life of mango fruit. Nevertheless, several limitations remain in the current literature. Most reported systems function as edible coatings or direct-contact films that adhere to fruit surfaces, altering appearance, complicating standardized application, and limiting scalability (Moradinezhad et al., 2025). The release behavior of encapsulated polyphenols is rarely quantified through kinetic modeling, which makes it difficult to predict the effective protective duration or to rationally optimize matrix design, despite the availability of well-established diffusion and empirical frameworks (Zhang et al., 2025). Furthermore, the translation of these laboratory-scale systems into commercial practice remains constrained by limited multi-fruit validation and the absence of direct comparisons with commercially established benchmark products (Krishnan et al., 2025).

*Poria cocos* polysaccharide (PCP) is a water-soluble β-D-glucan extracted from the sclerotium of *Wolfiporia cocos*, an edible and medicinal fungus with a long history of safe consumption in East Asia. PCP possesses favorable film-forming ability, hygroscopicity, and inherent bioactivity, and has been investigated in wound dressing, drug delivery, and functional food applications (Ding et al., 2025; Yang et al., 2024). Its potential as a structural matrix for controlled-release active packaging, however, remains largely unexplored. Our group previously demonstrated that annealing-induced hydrogen-bond rearrangement yields physically cross-linked PCP-tannic acid (TA) hydrogels with sustained polyphenol release and favorable mechanical integrity (Gao et al., 2026). However, the relatively dense annealed network restricts moisture infiltration and offers limited control over the release rate of encapsulated active agents. Introducing engineered porosity offers a direct route to overcome this constraint. Recently, salt porogen leaching has emerged as a straightforward approach to create interconnected porous architectures in polysaccharide hydrogels for food packaging (Liang et al., 2026). Yu et al. (2024) constructed sponge-like porous poly (vinyl alcohol)/chitosan hydrogels through freeze-casting combined with salting-out treatment, and demonstrated that the hierarchical pore structure enabled rapid molecular transport, sustained release of antimicrobial agents, and effective cushioning for kiwifruit preservation, highlighting the value of engineered porosity in active packaging matrices. Such interconnected pore channels facilitate moisture ingress and accelerate active agent transport relative to dense, non-porous networks, and the resulting release profiles can be quantified by the Korsmeyer-Peppas model, in which the diffusional exponent n captures the transition from Fickian diffusion to anomalous transport as pore architecture and network tortuosity vary (Hou et al., 2024; Qiu et al., 2024). Accordingly, we hypothesized that combining salt-leaching porosity with gradient annealing would yield PCP beads whose tunable pore architecture regulates moisture-triggered TA diffusion into the package headspace for residue-minimizing fruit preservation.

In the present study, porous PCP/TA composite hydrogel beads (PTBs) with three distinct pore architectures were fabricated through sodium chloride porogen leaching combined with a gradient annealing protocol. The resulting beads were characterized in terms of morphology, porosity, mechanical properties, and TA loading. Release kinetics were analyzed using the Korsmeyer-Peppas model to characterize the transport mechanism, and antioxidant efficacy was evaluated by headspace and direct-contact DPPH assays to link TA release with radical-scavenging activity. The preservation performance was evaluated on table grape and cherry tomato over 14 days, with a commercial sulfur dioxide-generating pad included as a positive control. The oral biocompatibility of the optimal formulation was assessed through a 28-day mouse feeding study with histological and serum biochemical analysis.

## 2 Materials and methods

### 2.1 Materials

*Poria cocos* β-glucan (PCP, purity ≥95%, weight-average molecular weight 8–10 × 10^4^ g/mol, monosaccharide composition with glucose accounting for over 95%) was supplied by Shanghai Winherb Medical Technology Co., Ltd. (Shanghai, China). Tannic acid (TA, analytical grade), dimethyl sulfoxide (DMSO, analytical grade), sodium chloride (NaCl, analytical grade), 2,2-diphenyl-1-picrylhydrazyl (DPPH), Folin-Ciocalteu reagent, and gallic acid standard were procured from Shanghai Macklin Biochemical Co., Ltd. (Shanghai, China). All remaining reagents were of analytical grade and used without additional purification. Luria-Bertani (LB) agar and broth were obtained from Qingdao Hope Bio-Technology Co., Ltd. (Qingdao, China). *Escherichia coli* (ATCC 25922) and *Staphylococcus aureus* (ATCC 25923) were provided by the Guangdong Microbial Culture Collection Center (Guangzhou, China). Commercial dual-release sulfur dioxide pads (UVASYS) were purchased from Shandong Huaxu Agricultural Technology Co., Ltd. (Shandong, China). Red Globe grapes (*Vitis vinifera* cv. Red Globe) and cherry tomatoes (*Solanum lycopersicum* cv. Shengnü) at commercial maturity (Red Globe: full red-purple color with ≥18% soluble solids; Shengnü cherry tomato: ≥80% red surface, hue angle ≈40°) were acquired from a wholesale market in Xiaogan, China, on the day of each experiment, with fruit of uniform size, color, and free from visible mechanical injury selected for use. Female Institute of Cancer Research (ICR) mice (SPF grade, 6–8 weeks old) were purchased from Shulaibao Biotechnology Co., Ltd. (Wuhan, China).

### 2.2 Fabrication of porous hydrogel beads

Porous PCP/TA hydrogel beads were prepared via a thermal dissolution, extrusion, and gradient annealing procedure, as illustrated in Fig. 1A. PCP was dissolved in DMSO at 3, 4, or 5% (*w*/*v*) under magnetic stirring at 80 °C, followed by addition of TA at a fixed concentration of 2% (*w*/*v*) to each solution. NaCl was then incorporated as a porogen at a fixed concentration of 10% (*w*/*v*) in all formulations, after being sieved to a particle size range of 75–150 μm. The resulting hot solution was extruded dropwise through a 21-gauge needle into cylindrical molds to generate uniform droplets. Gradient annealing from 80 to 25 °C was applied to induce physical gelation and stabilize the bead architecture. The gelled beads were repeatedly rinsed with deionized water to remove NaCl and residual DMSO until the conductivity of the washing effluent reached that of deionized water; residual DMSO in the final beads was additionally verified below the ^1^H NMR detection limit (Method S1), after which the beads were stored in deionized water for subsequent use. Beads containing 4% PCP without TA were designated PB, whereas those loaded with TA at 3, 4, and 5% PCP were designated PTB-1, PTB-2, and PTB-3, respectively.

**Fig. 1:**
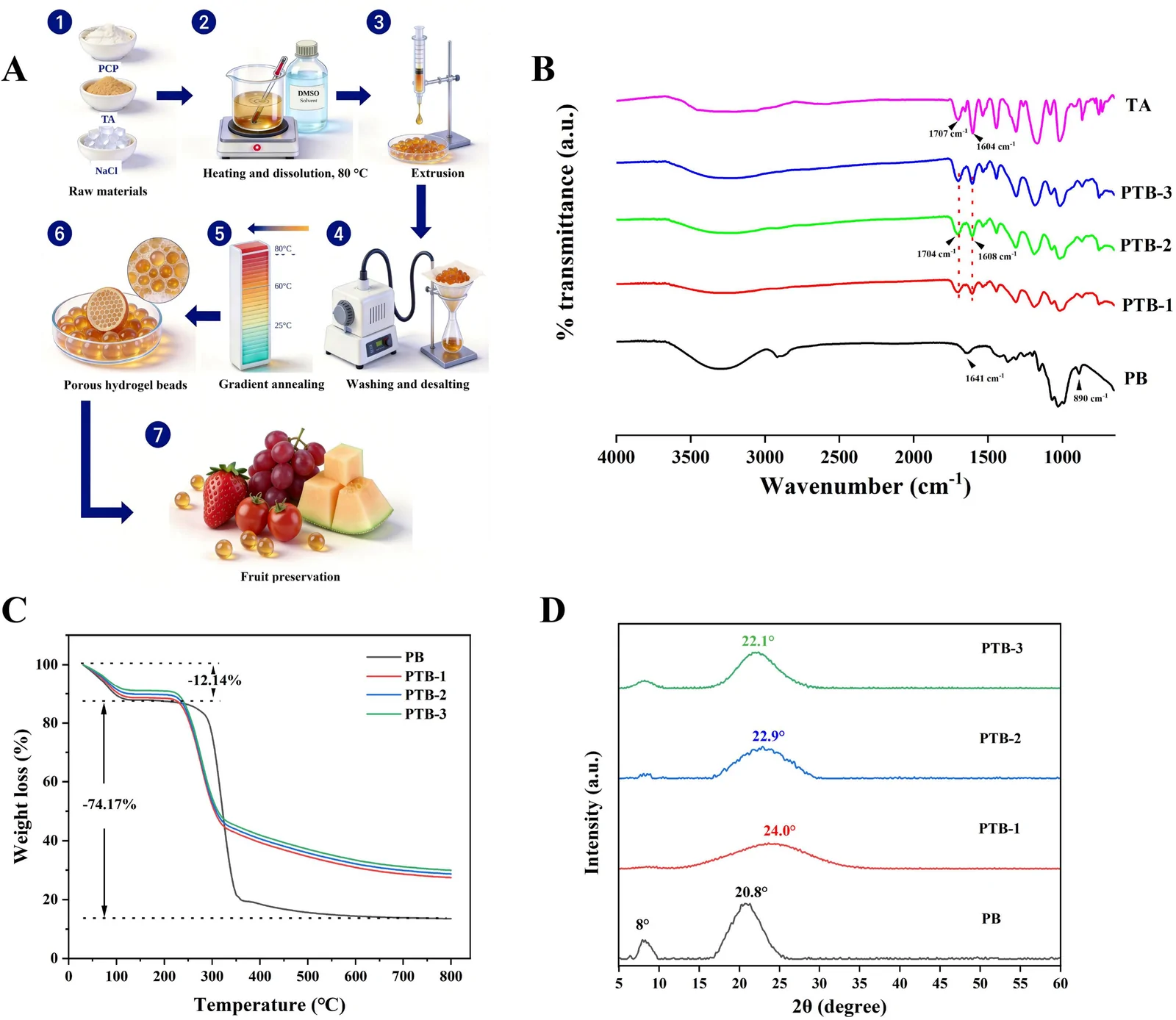
Fabrication and structural characterization of porous TA-loaded hydrogel beads. (A) Schematic illustration of the fabrication process, including raw material preparation, thermal dissolution at 80 °C, extrusion, gradient annealing at 80 to 25 °C, washing and desalting, and the resulting porous hydrogel beads applied to fruit preservation. (B) FTIR spectra of TA, PB, and PTB-1 to PTB-3. (C) TGA curves of PB and PTB-1 to PTB-3. (D) XRD patterns of PB and PTB-1 to PTB-3.

### 2.3 Structural characterization

Fourier transform infrared (FTIR) spectra were acquired on a Nicolet iS50 spectrometer (Thermo Fisher Scientific, Waltham, MA, USA) with the KBr pellet method over 4000–400 cm^−1^ at 4 cm^−1^ resolution and 32 cumulative scans. Thermogravimetric analysis (TGA) was carried out on a Mettler Toledo TGA2 system (Greifensee, Switzerland) under a nitrogen atmosphere at a flow rate of 50 mL/min, heating from 30 to 800 °C at 10 °C/min. X-ray diffraction (XRD) patterns were obtained using a D8 Advance diffractometer (Bruker, Karlsruhe, Germany) with Cu Kα radiation (λ = 1.5406 Å) at 40 kV and 40 mA, scanning 5 to 60° (2θ) in 0.02° increments.

### 2.4 Morphology and porosity

Cross-sectional morphology was examined by scanning electron microscopy (SEM) using an SU8010 instrument (Hitachi, Tokyo, Japan). Freeze-dried beads were cryo-fractured in liquid nitrogen, sputter-coated with gold, and imaged at an accelerating voltage of 15 kV. Pore size distributions were quantified from SEM micrographs with ImageJ software (NIH, Bethesda, MD, USA), measuring at least 100 pores per sample. Total porosity was determined by the liquid displacement method with absolute ethanol as the immersion medium. Sphericity was derived from digital images as the ratio of the minimum to maximum Feret diameter with 50 beads analyzed per group (Zhao et al., 2026). Bead formation yield was calculated according to Eq. (1):

(1) $$\text{Yield}\;\left(\%\right)=\frac{N_{i}}{N_{t}}\times 100$$

where N_i_ is the number of intact, non-coalesced spherical beads recovered after washing and N_t_ is the total number of extruded droplets (*n* = 3).

### 2.5 Rheological and mechanical properties

Rheological measurements were conducted on a DHR-2 rheometer (TA Instruments, New Castle, DE, USA) fitted with a 20 mm parallel-plate geometry at a 1 mm gap. Frequency sweeps were performed over 0.1–10 Hz at a constant strain of 1% within the linear viscoelastic region at 25 °C, and the storage modulus (G′) at 1 Hz was recorded. Compressive testing was performed on an Instron 5967 universal testing machine (Instron, Norwood, MA, USA) equipped with a cylindrical probe. Hydrated beads were compressed to 60% strain at a crosshead speed of 1 mm/s, and the compressive modulus was derived from the slope of the linear region between 5 and 15% strain (*n* = 5). Handling robustness was evaluated by tweezer lifting and manual compression with full release in water.

### 2.6 Moisture sorption isotherms

Moisture sorption was measured by a static gravimetric method at 25 °C (Dordevic et al., 2023). Saturated solutions of MgCl₂, NaBr, NaCl, and KNO₃ were used to maintain relative humidity levels of 33, 57, 75, and 93%, while deionized water provided 100% RH. Freeze-dried beads weighing 0.5 g were placed in each desiccator and weighed at regular intervals until the mass change over 24 h was below 0.001 g. Desorption isotherms were obtained by transferring fully hydrated beads through progressively lower humidity levels. Equilibrium moisture content was expressed as grams of water per 100 g dry weight (*n* = 3).

### 2.7 TA release kinetics

TA release was evaluated in parallel in 50 mL of phosphate-buffered saline (PBS, pH 7.4) and 0.1 M citrate buffer (pH 4.0) at 25 °C with continuous shaking at 100 rpm. Hydrated beads weighing 0.5 g were sealed in a dialysis membrane with a molecular weight cutoff of 8–14 kDa and immersed in the release medium. At each sampling time point of 0.25, 0.5, 1, 2, 4, 8, 12, 24, 36, 48, and 72 h, a 1 mL aliquot was withdrawn and replenished with an equal volume of the corresponding medium; a pH-matched blank without beads was subtracted to account for Folin-Ciocalteu background. Because the 8–14 kDa membrane is freely permeable to TA (MW 1701 Da), the profiles quantify bead-to-medium diffusion and are used for inter-formulation comparison. The TA concentration was quantified by the Folin-Ciocalteu method using gallic acid as the standard, and absorbance was read at 760 nm on a UV-1800 spectrophotometer (Shimadzu, Kyoto, Japan). Actual TA content of the beads was quantified by methanol extraction and the Folin-Ciocalteu assay (Method S2), and cumulative release was normalized to the measured loaded TA (*n* = 3). The release mechanism was examined by fitting early-phase data where Mt./M∞ < 0.6 to the Korsmeyer-Peppas model (Eq. 2):

(2) $$\frac{M_{t}}{M_{\infty }}={\mathrm{kt}}^{n}$$

where Mt/M∞ is the fractional release, k is the release rate constant, and n is the release exponent.

### 2.8 Antioxidant activity

A headspace DPPH assay was established based on the method described by Brand-Williams et al. (1995), with modification to evaluate non-contact antioxidant activity. DPPH ethanol solution at 0.1 mM was dispensed in 3 mL aliquots at the bottom of sealed glass vials, and 0.2 g of hydrated beads was supported on a perforated polypropylene mesh fixed in the upper half of the sealed vial, remaining physically separated from the 3 mL DPPH solution throughout incubation. Following incubation at 25 °C in the dark for 1, 2, 4, 8, 12, 24, and 48 h, absorbance was measured at 517 nm using a microplate reader (Tecan Infinite M200, Männedorf, Switzerland). A direct-contact assay was performed in parallel by immersing beads in 3 mL of DPPH solution with absorbance recorded at identical time points. Vials without beads and vials containing PB beads without TA served as controls. DPPH radical scavenging activity was calculated according to Eq. (3):

(3) $$\text{Scavenging activity}\;\left(\%\right)=\frac{A_{0}-A_{S}}{A_{0}}\times 100$$

where A₀ and Aₛ are the absorbance of the control and sample, respectively. The correlation between total phenolic release and DPPH scavenging was evaluated by linear regression.

### 2.9 Antibacterial activity

Antibacterial activity was evaluated against *S. aureus* and *E. coli* by a broth co-culture method. Bacterial suspensions were adjusted to 10^6^ CFU/mL in LB broth, and 0.1 g of PTB-2 beads was added to 5 mL of the inoculated broth. Cultures without beads and cultures containing PB beads of equivalent PCP mass served as the blank and TA-free controls, respectively. After incubation at 37 °C with shaking at 150 rpm, 200 μL of each co-culture was transferred to a 96-well plate, and optical density at 600 nm (OD₆₀₀) was measured using a microplate reader at 0, 6, 12, 18, and 24 h. The bacterial inhibition rate was calculated according to Eq. (4):

(4) $$\text{Inhibition rate}\;\left(\%\right)=\frac{{\mathrm{OD}}_{c}-{\mathrm{OD}}_{s}}{{\mathrm{OD}}_{c}}\times 100$$

where OD_c_ and OD_s_ are the absorbance values of the blank control and PTB-2 co-culture, respectively. For visual confirmation, 100 μL of each co-culture was spread onto LB agar plates after 24 h and photographed.

### 2.10 Soil burial degradation

Freeze-dried bead samples of known initial weight W₀ were sealed in polyethylene mesh bags with 1 mm pores and buried in garden soil at a depth of 5 cm. Soil temperature was maintained at 25 °C and moisture content at 40–50%. On days 7, 14, 21, 28, 35, 42, 49, and 56, the samples were retrieved, rinsed with deionized water, lyophilized, and weighed to obtain Wt. Morphological changes were recorded photographically at each time point. Weight loss was calculated according to Eq. (5):

(5) $$\text{Weight loss}\;\left(\%\right)=\frac{W_{0}-W_{t}}{W_{0}}\times 100$$

where W₀ and Wt are the dry weights before burial and after retrieval, respectively.

### 2.11 Whole fruit preservation

Red Globe grape clusters weighing 200 g and cherry tomatoes weighing 150 g per replicate were randomly allocated to three treatment groups: blank control without beads or pads, PTB-2 with 5 g hydrated beads per container, and UVASYS with one dual-release SO₂ pad per container following the manufacturer's recommendation. Fruit were packed in perforated polyethylene clamshells (16 × 12 × 6 cm; internal volume 1100 mL; headspace 380 mL above the fruit) bearing six circular perforations (4 mm i.d.) on the lid. The 5 g hydrated-bead dose was the lowest amount maintaining 85–95% RH and measurable headspace antioxidant activity in preliminary trials, whereas one UVASYS pad, placed at the opposite corner, followed the manufacturer's charge for this package size; beads were held on an elevated mesh tray without fruit contact. Packages were stored at 25 °C for 14 days, representing the ambient unrefrigerated retail window of both commodities as established in preliminary trials.

Quality parameters were assessed at 2-day intervals. Weight loss was expressed as a percentage relative to the initial weight. Decay incidence was determined as the proportion of berries or fruits exhibiting visible fungal growth or tissue breakdown (Yu et al., 2025). Firmness was measured with a Universal TA texture analyzer (Shanghai Tengba Instrument Technology Co., Ltd., Shanghai, China) fitted with a 2 mm cylindrical probe for grapes or a flat probe for tomatoes and expressed in Newtons. For grapes, total anthocyanin was extracted with 1% HCl in methanol and quantified by the pH differential method (Giusti & Wrolstad, 2001), with results expressed as mg cyanidin-3-glucoside equivalents per 100 g fresh weight. Stem browning was graded on a 0 to 4 scale from completely green to completely brown and reported as a browning index (Qi & Jiang, 2026). For tomatoes, lycopene was extracted with a hexane-ethanol-acetone mixture at a 2:1:1 volume ratio and quantified spectrophotometrically at 503 nm in mg per 100 g fresh weight (Fish et al., 2002). Surface color was measured with a CR-400 colorimeter, and the hue angle was calculated. Each treatment was performed in triplicate. TA on fruit surfaces and in the flesh at days 7 and 14 was quantified by rinse/extraction followed by the Folin-Ciocalteu assay against a TA calibration, with matrix-matched blanks and spike-recovery controls (Method S3).

### 2.12 In vivo biosafety evaluation

All protocols for animal experiments presented in this paper have been submitted to and reviewed by the Ethics Committee of Hubei Engineering University (protocol code: A2026010), ensuring strict compliance with the ethical guidelines and procedural frameworks established by the National Research Council's Guide for the Care and Use of Laboratory Animals.

Because the beads are designed as a non-contact headspace generator and are not intended for ingestion, this study was conceived as a worst-case assessment of accidental whole-bead ingestion rather than a simulation of packaging use, with 2 g/kg representing an exaggerated daily intake relative to any plausible package transfer. Twenty ICR mice were acclimatized for one week and then randomly divided into two groups (*n* = 10). The PTB-2 group received 2 g/kg body weight of homogenized PTB-2 beads by oral gavage daily for 28 days, while the control group received an equal volume of saline. Body weight was recorded every two days. At day 28, blood samples were collected for serum biochemical analysis, including alanine aminotransferase (ALT), aspartate aminotransferase (AST), alkaline phosphatase (ALP), blood urea nitrogen (BUN), creatinine (CREA), total protein (TP), albumin (ALB), and glucose (GLU). Major organs (heart, liver, spleen, lung, and kidney) were harvested, weighed for organ index calculation, fixed in 4% paraformaldehyde, sectioned, and stained with hematoxylin and eosin (H&E) for histopathological examination.

### 2.13 Statistical analysis

All data are expressed as mean ± SD from at least three independent experiments. Single-endpoint data (loading, porosity, mechanics, sorption) were compared by one-way ANOVA with Tukey's test. Measurements repeated over time on the same fabrication batch, culture flask, or package (TA release, bacterial growth, fruit quality) used two-way repeated-measures ANOVA with treatment and time as fixed factors; independently sampled soil-burial data used two-way factorial ANOVA. The experimental unit was the independent fabrication batch, culture flask, or package. Mauchly's test was applied, with Greenhouse–Geisser correction when sphericity was violated, followed by Tukey's test at individual time points after a significant interaction. Analyses used SPSS 26.0 (*p* < 0.05); curve fitting used OriginPro 2024.

## 3 Results and discussion

### 3.1 Fabrication and structural characterization of the PCP/TA porous hydrogel beads

The porous hydrogel beads were prepared through a multi-step route involving thermal dissolution of PCP, TA, and NaCl in DMSO at 80 °C, extrusion into uniform droplets, gradient annealing from 80 to 25 °C, and washing for salt removal (Fig. 1A). NaCl served as a porogen that was subsequently leached during washing, while temperature-gradient annealing stabilized the resulting porous architecture.

FTIR spectroscopy confirmed that TA was successfully integrated into the PCP network (Fig. 1B). Pure TA displayed characteristic peaks at 1707 and 1614 cm^−1^, assigned to ester carbonyl stretching and aromatic C=C vibration, respectively. PB showed a broad band at 1641 cm^−1^ together with a peak at 890 cm^−1^. All PTB formulations exhibited two new peaks at 1704 and 1608 cm^−1^ that were absent in PB. The 1704 cm^−1^ band coincided with the carbonyl signal of TA, whereas the aromatic C=C vibration shifted from 1614 to 1608 cm^−1^ (Gao et al., 2026). These shifts reflected the formation of intermolecular hydrogen bonds between the phenolic hydroxyl groups of TA and the PCP backbone, demonstrating that TA was molecularly incorporated rather than simply admixed. A hydrogen-bonded network facilitates homogeneous TA distribution and supports sustained release throughout the storage period.

TGA demonstrated that TA incorporation improved the thermal stability of the beads, while varying PCP concentration produced only minor differences attributable to initial moisture content (Fig. 1C). PB exhibited a moisture loss of 12.14% below 200 °C followed by a major degradation of 74.17%, leaving 13.69% residue at 800 °C. All PTB formulations showed substantially lower moisture loss ranging from 8.95% in PTB-3 to 11.38% in PTB-1, with PTB-2 at an intermediate value of 10.18%. The three PTB formulations exhibited comparable degradation losses of 60.8% and char yields between 27.76% and 30.25% at 800 °C, confirming that the porogen leaching process did not alter the chemical composition of the PCP-TA network. The char yield of PTB-2 was more than twice that of PB, indicating that hydrogen bonding between PCP and TA restricted chain mobility while the phenolic moieties acted as thermal barriers that promoted char formation and suppressed rapid depolymerization (Qin et al., 2022).

XRD analysis revealed that TA incorporation transformed the semi-crystalline PCP structure into a predominantly amorphous network, with the degree of crystallinity dependent on PCP concentration (Fig. 1D). PB displayed two well-defined diffraction peaks at 8° and 20.8°, indicative of ordered chain packing. PTB-3 at 5 wt% PCP retained a relatively sharp peak at 22.1° with a discernible 8° shoulder, whereas PTB-2 at 4 wt% and PTB-1 at 3 wt% PCP exhibited progressively broadened halos centered at 22.9° and 24.0°, accompanied by gradual attenuation of the low-angle signal. The monotonic peak shift to higher angles and peak broadening from PTB-3 to PTB-1 reflected decreasing crystalline ordering at lower PCP content. Comparison of PB and PTB-2, both prepared at 4 wt% PCP, further indicated that TA molecules intercalated between PCP chains and established hydrogen bonds that disrupted ordered rearrangement. This amorphous architecture favors water absorption and active compound diffusion, both of which are beneficial for maintaining a hydrated fruit surface and delivering antioxidant TA during preservation (Tanpichai et al., 2025).

### 3.2 Morphology and moisture sorption behavior

The beads were evaluated for morphology and moisture sorption performance (Fig. 2). TA incorporation substantially improved bead formation: sphericity increased from 0.72 in PB to 0.87 in PTB-2, and formation yield from 21.9% to 58.1%, with intermediate values for PTB-1 and PTB-3 (Fig. 2B and C). This indicated that TA-mediated hydrogen bonding reinforced the extruded droplets during gradient annealing, minimizing deformation and coalescence. Moisture sorption isotherms displayed a Type II isotherm with gradual uptake below 75% RH and a pronounced increase above 93% RH, reflecting capillary condensation within the porous network. At 100% RH, equilibrium moisture contents reached 242.36 ± 5.37, 225.71 ± 5.06, 134.52 ± 5.24, and 103.91 ± 4.18 g per 100 g dry weight for PTB-2, PTB-3, PB, and PTB-1, respectively. The ranking was consistent at 93% RH, where PTB-2 retained 101.64 ± 1.80 g per 100 g compared with 92.58 ± 1.71 for PTB-3, 85.49 ± 1.77 for PB, and 79.14 ± 2.75 for PTB-1. A clear hysteresis between adsorption and desorption branches indicated that a fraction of absorbed water was strongly bound within the polymer matrix and not readily released. This uptake is consistent with superabsorbent polysaccharide hydrogels (Safwat et al., 2025), and far exceeds the 38–49 g/100 g of pure polysaccharide powders at 85% RH (Miyauchi, 2023), confirming that the porous architecture enhanced vapor capture relative to non-porous matrices.

**Fig. 2:**
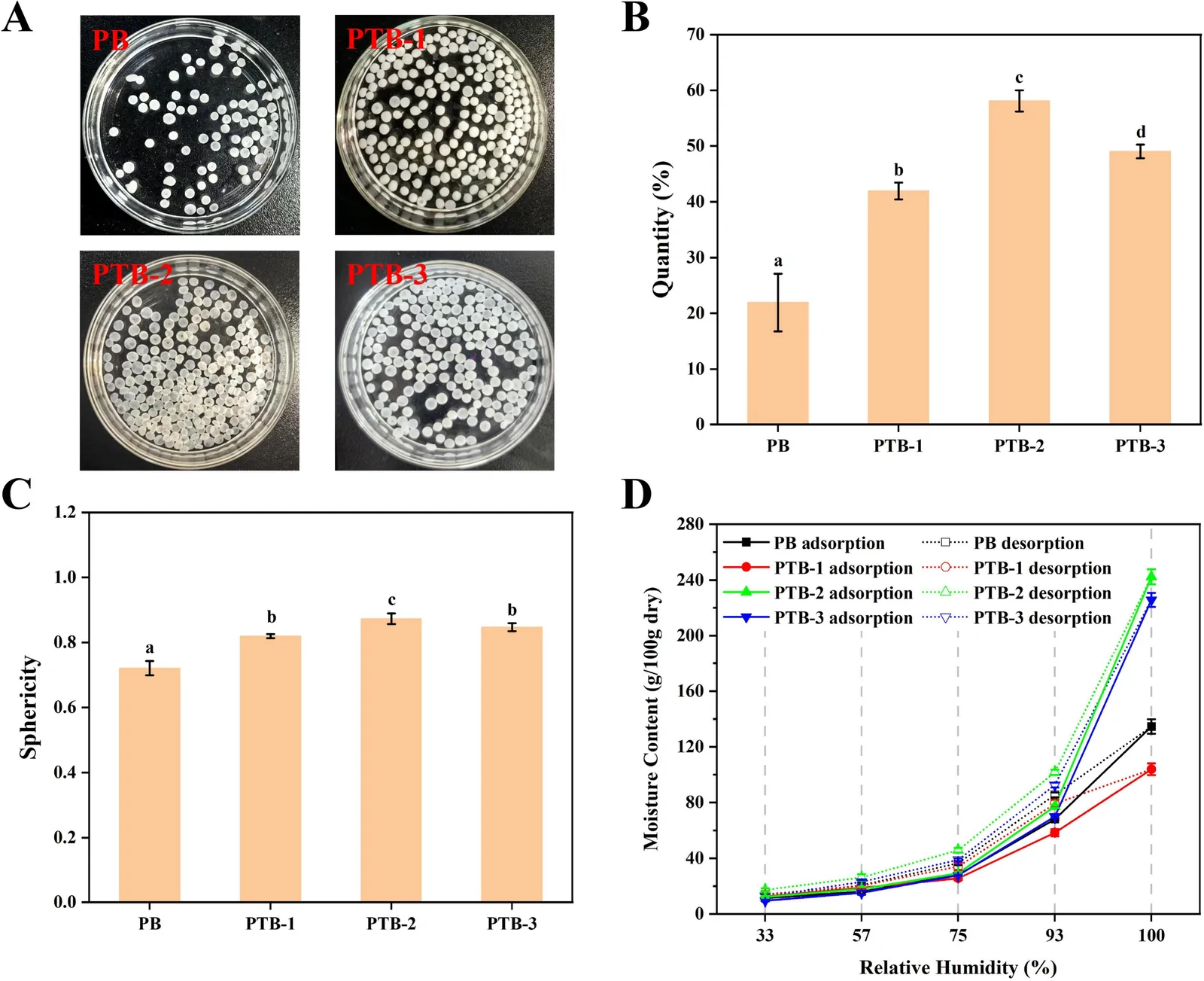
Morphology and moisture sorption properties of hydrogel beads. (A) Digital photographs of PB, PTB-1, PTB-2, and PTB-3. (B) Bead formation yield. (C) Sphericity. (D) Moisture adsorption and desorption isotherms at 33, 57, 75, 93, and 100% relative humidity. Data are presented as mean ± SD. Different letters indicate significant differences at *p* < 0.05.

Notably, moisture uptake did not scale linearly with total pore volume. Among the TA-loaded formulations, PTB-1 with the largest pores absorbed the least moisture, whereas PTB-2 with intermediate porosity achieved the highest uptake. This trend reflected an optimal balance between capillary retention and void volume: the coarse, less uniform channels in PTB-1 favored rapid drainage over sustained retention, while the finer pore network of PTB-2 generated stronger capillary forces without sacrificing storage capacity (Nafar et al., 2026). PTB-3 was constrained by limited total pore volume despite its strongest capillary action. PB, despite its largest pores, absorbed less than PTB-2 because the loosely crosslinked TA-free network retained water mainly through macropore filling rather than capillary condensation. The convergence of maximum sphericity, highest yield, and greatest moisture sorption in PTB-2 identified this formulation as the most effective for regulating the humid microenvironment surrounding stored fruit.

### 3.3 Microstructural architecture and porosity

SEM imaging revealed that all beads possessed an interconnected porous network, but TA incorporation progressively refined the pore architecture (Fig. 3A). PB exhibited large, irregular macropores with a mean diameter of 50.32 ± 3.31 μm, whereas PTB-1, PTB-2, and PTB-3 displayed progressively smaller and more uniform pores of 36.35 ± 1.01, 31.21 ± 2.95, and 24.93 ± 1.72 μm, respectively (Fig. 3B). The broad and skewed pore size distribution of PB reflected uncontrolled phase separation during annealing without TA-mediated crosslinking. Increasing PCP concentration shifted the distributions toward narrower profiles centered at smaller diameters, consistent with the SEM observations of a denser and more homogeneous honeycomb-like structure in PTB-2 and PTB-3.

**Fig. 3:**
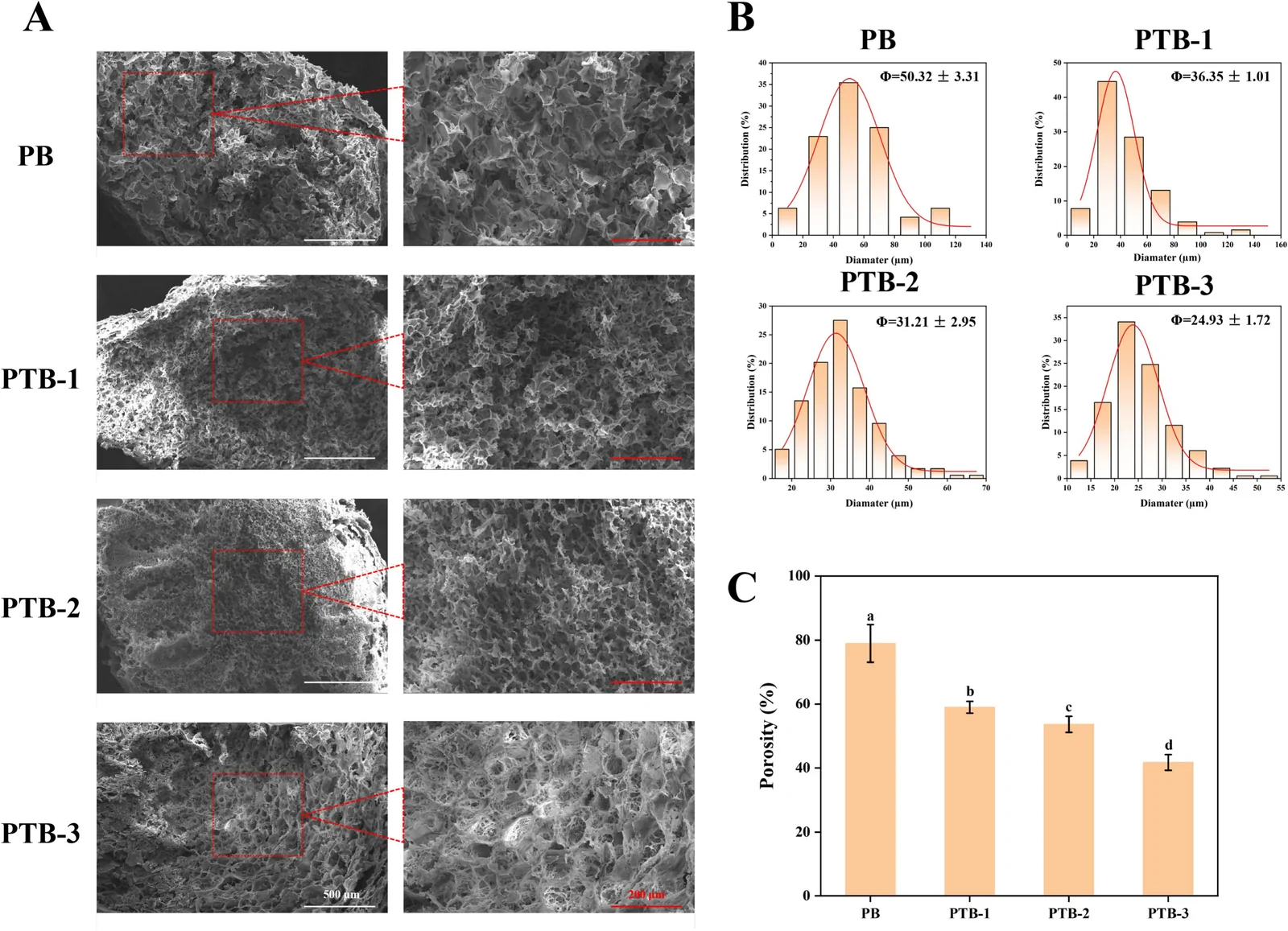
Microstructural characterization of hydrogel beads. (A) Cross-sectional SEM images at low and high magnification. Scale bars: 500 μm and 200 μm. (B) Pore size distribution histograms with Gaussian fits. (C) Porosity of PB, PTB-1, PTB-2, and PTB-3. Data are presented as mean ± SD. Different letters indicate significant differences at *p* < 0.05.

Porosity followed the same decreasing trend. PB showed the highest porosity at 78.99 ± 5.90%, while PTB-1, PTB-2, and PTB-3 recorded 58.99 ± 1.90, 53.61 ± 2.53, and 41.70 ± 2.46%, respectively (Fig. 3C). The coordinated reduction in pore diameter and porosity was attributed to the increasing density of intermolecular hydrogen bonds between PCP and TA. A higher PCP concentration increased chain entanglement during gradient annealing, restricting pore expansion and producing a more compact crosslinked matrix (Zhou et al., 2022).

The progressive refinement of pore architecture from PB to PTB-3 was consistent with the mechanical data, where increasing PCP concentration produced denser and more rigid networks. Among the TA-loaded formulations, PTB-2 achieved the most favorable balance of pore size, interconnectivity, and total void volume, providing a structural basis for its superior moisture retention and mechanical integrity observed in subsequent evaluations.

### 3.4 Rheological and mechanical properties

Rheological frequency sweeps confirmed that all formulations formed stable elastic gel networks, with G' consistently exceeding G" across 0.1 to 10 Hz (Fig. 4A). The storage modulus of PB remained near 400 Pa throughout the tested range, while TA-loaded beads showed markedly higher values. PTB-2 exhibited the highest G', followed by PTB-3 and PTB-1. The relative invariance of G' with frequency indicated that the hydrogen-bonded PCP/TA networks were structurally stable rather than transient (Zhong et al., 2024). The loss modulus G" remained below 150 Pa for all groups, confirming predominantly elastic rather than viscous behavior.

**Fig. 4:**
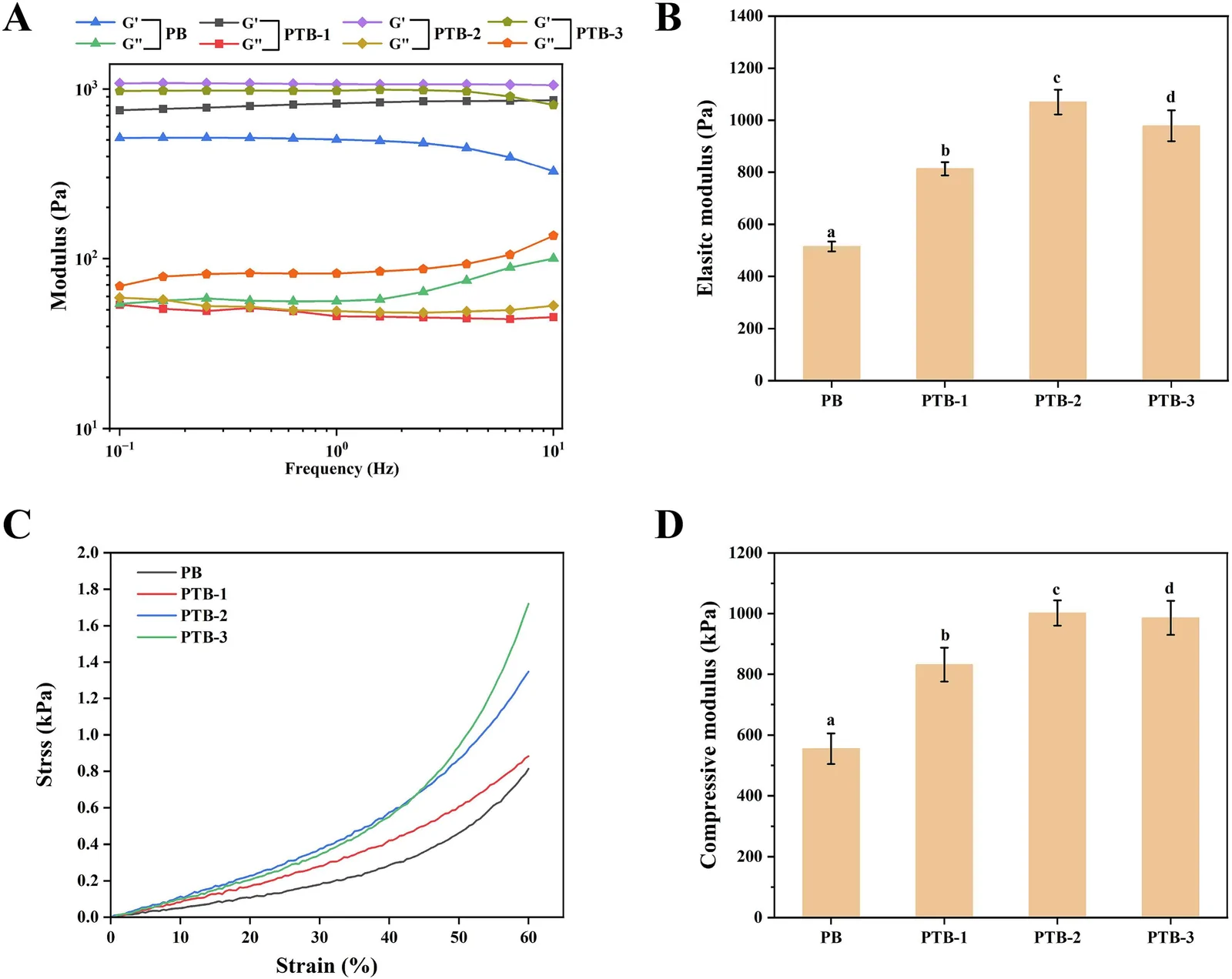
Rheological and mechanical characterization of hydrogel beads. (A) Frequency sweep of storage modulus G' and loss modulus G" from 0.1 to 10 Hz. (B) Elastic modulus. (C) Compressive stress-strain curves from 0 to 60% strain. (D) Compressive modulus. Data are presented as mean ± SD. Different letters indicate significant differences at *p* < 0.05.

The elastic modulus quantified from the linear viscoelastic region followed the same trend. PB recorded 514.90 ± 19.06 Pa, increasing to 813.35 ± 26.06 Pa for PTB-1 and reaching a maximum of 1070.24 ± 47.64 Pa for PTB-2 (Fig. 4B). PTB-3 showed a slightly lower value of 978.77 ± 59.20 Pa. The nearly twofold increase from PB to PTB-2 demonstrated that TA-mediated hydrogen bonding substantially reinforced the network (Zhao et al., 2025). The modest decline from PTB-2 to PTB-3 suggested that higher PCP concentration produced a more densely packed but somewhat less efficiently load-bearing structure, consistent with the lower porosity and reduced pore interconnectivity observed in SEM.

Compressive stress-strain curves displayed a characteristic J-shaped profile with an initial linear region followed by steep strain hardening above 40% strain (Fig. 4C). The compressive modulus calculated from the 5 to 15% linear region was 554.71 ± 50.22 Pa for PB, rising to 832.08 ± 56.15 Pa for PTB-1, 1002.33 ± 41.64 Pa for PTB-2, and 986.27 ± 55.89 Pa for PTB-3 (Fig. 4D). The close agreement between rheological and compressive modulus values validated that both measurement modes probed the same crosslinked network. At strains above 50%, PTB-3 exhibited the steepest stress rise, reaching 1.72 kPa at 60% strain compared with 1.36 kPa for PTB-2. This late-stage crossover reflected the lower porosity of PTB-3, which densified more rapidly under compression as pore walls came into contact (Guo et al., 2026). For fruit preservation applications, the balanced stiffness and deformability of PTB-2 were advantageous, as the beads could conform to fruit surfaces while maintaining structural integrity during handling and storage. Beads withstood tweezer lifting and manual compression–release without rupture or permanent fragmentation (Fig. S3), confirming integrity sufficient for in-package handling.

### 3.5 TA release kinetics and antioxidant activity

The in vitro release of TA from PTB-1, PTB-2, and PTB-3 was monitored over 72 h in parallel in PBS (pH 7.4) and 0.1 M citrate buffer (pH 4.0), the latter approximating the acidic surface pH of both fruits (Fig. 5A, B). In PBS, all formulations showed a biphasic profile, with a 4 h burst followed by a plateau after 48 h: PTB-1, PTB-2, and PTB-3 released 60.25 ± 0.48%, 55.32 ± 0.78%, and 31.99 ± 0.40% at 4 h and 93.55 ± 0.53%, 97.43 ± 0.06%, and 89.56 ± 0.72% at 72 h. The burst tracked pore architecture: the coarse large-pore network of PTB-1 admitted water rapidly but left wall-embedded TA unreached, the dense PTB-3 matrix excluded deeply entrapped TA, and PTB-2 best combined interconnectivity with accessible surface area, giving the most complete extraction. All values were normalized to measured bead TA content (Fig. S2). Loading content and encapsulation efficiency were 29.26 ± 2.22% and 60.06 ± 1.89% for PTB-1, 37.02 ± 3.26% and 88.74 ± 1.47% for PTB-2, and 40.18 ± 2.15% and 90.21 ± 2.71% for PTB-3, increasing with PCP content as denser hydrogen-bonded networks retained more TA. On a TA-feed basis, 72 h release accounted for 56.2, 86.5, and 80.8% of fed TA, identifying PTB-2 as the optimal retention–release balance. Release at pH 4.0 closely matched PBS, with 4 h bursts of 54.92 ± 1.95%, 51.93 ± 2.78%, and 28.85 ± 2.01% and 72 h values of 92.95 ± 1.22%, 97.08 ± 2.08%, and 88.32 ± 4.23% for PTB-1, PTB-2, and PTB-3, within 1.3 percentage points of PBS at the plateau. The modest early retardation is consistent with protonation of TA galloyl groups strengthening TA–PCP hydrogen bonding, without reducing total extractable TA, indicating that the release behavior established in PBS remains valid on acidic fruit surfaces.

**Fig. 5:**
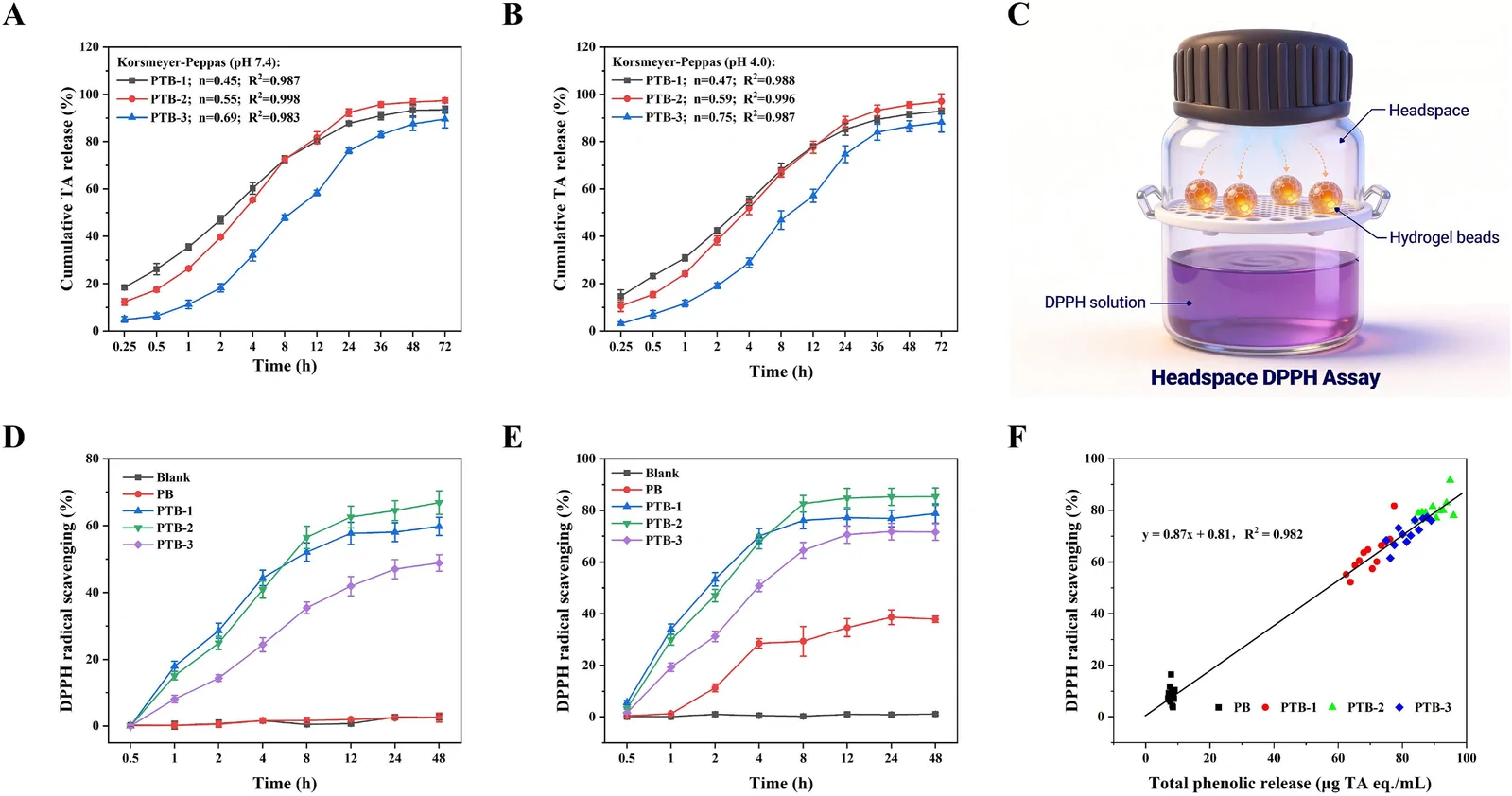
TA release kinetics and antioxidant activity of PTBs. (A) Cumulative TA release over 72 h in PBS (pH 7.4), with Korsmeyer–Peppas n and R^2^ values indicated. (B) Cumulative TA release over 72 h in citrate buffer (pH 4.0), with Korsmeyer–Peppas n and R^2^ values indicated. (C) Schematic of the headspace DPPH assay setup. (D) DPPH radical scavenging under headspace conditions over 48 h. (E) DPPH radical scavenging under direct-contact conditions over 48 h. (F) Correlation between total phenolic release and DPPH scavenging fitted by linear regression. Data are presented as mean ± SD (*n* = 3).

Korsmeyer–Peppas fits were excellent in both media (R^2^ ≥ 0.983). In PBS, n rose from 0.45 (PTB-1) to 0.55 (PTB-2) and 0.69 (PTB-3), and at pH 4.0 from 0.47 to 0.59 and 0.75, while k decreased from 0.37 to 0.27 and 0.13 in PBS and from 0.32 to 0.25 and 0.12 in citrate buffer. The preserved monotonic increase in n shows that pore-governed Fickian-to-anomalous transition, rather than pH, sets the mechanism, with the densest PTB-3 network (41.7% porosity; 978.77 Pa, Fig. 4B) requiring the greatest matrix relaxation. The n range of 0.45–0.75 agrees with other crosslinked polysaccharide–polyphenol systems, including cellulose nanocrystal-alginate microspheres (*n* = 0.43–0.85) (Batta-Mpouma et al., 2025) and quercetin-loaded hyaluronic acid hydrogels (*n* = 0.53–0.96) (Su et al., 2025), confirming that non-Fickian transport governed by coupled diffusion and polymer relaxation is a common release mechanism in crosslinked polysaccharide networks.

A headspace DPPH assay was designed to evaluate whether the released TA could exert antioxidant protection without direct contact, simulating active packaging conditions where beads are separated from fruit surfaces (Fig. 5C). Blank and PB maintained negligible scavenging throughout 48 h, remaining below 2.66 ± 0.68% and 2.51 ± 1.34%, respectively. All TA-loaded beads showed time-dependent radical scavenging that tracked their release profiles (Fig. 5D). At 1 h, PTB-1 achieved 17.95 ± 1.48%, followed by PTB-2 at 15.12 ± 1.20% and PTB-3 at 8.04 ± 1.11%, reflecting the faster initial TA release from the more porous matrix. By 8 h, PTB-2 overtook PTB-1, reaching 56.47 ± 3.36% compared with 52.08 ± 2.75%. At 48 h, PTB-2 attained the highest scavenging of 66.85 ± 3.54%, while PTB-1 and PTB-3 reached 59.76 ± 2.68% and 48.84 ± 2.43%. The crossover between PTB-1 and PTB-2 paralleled the release kinetics, where the large pores of PTB-1 enabled fast initial TA discharge but depleted the available reservoir sooner, while the moderate pore network of PTB-2 sustained release over a longer period. The dense matrix of PTB-3 limited moisture-mediated TA transfer, yielding the lowest headspace activity. Given the negligible volatility of TA (MW 1701 Da), this activity is attributed to moisture-mediated transfer rather than volatilization: sorbed water (242 g/100 g at 100% RH) forms a TA-rich surface film whose condensed microdroplets reach the assay solution while beads remain physically separated on an elevated mesh, excluding direct contact.

Under direct contact, all TA-loaded beads exhibited substantially higher scavenging, with PTB-2 reaching 85.35 ± 3.40% at 48 h, followed by PTB-1 at 78.77 ± 3.74% and PTB-3 at 71.61 ± 3.18% (Fig. 5E). PB also showed moderate activity of 37.92 ± 1.21% after a 2 h lag, attributable to direct contact between PCP chains and DPPH radicals that did not occur in the headspace configuration. The consistently higher values under direct contact confirmed that moisture-mediated transfer through the headspace was the rate-limiting step in the headspace assay, while full immersion allowed both soluble TA and surface-exposed polyphenolic groups to react with DPPH radicals without a diffusion barrier.

A linear regression between total phenolic release and DPPH scavenging yielded y = 0.87× + 0.81 with R^2^ = 0.982 (Fig. 5F). The near-unity slope and strong correlation demonstrated that radical scavenging was quantitatively attributable to released TA rather than other matrix components. Data points for PB clustered near the origin, reinforcing that PCP alone contributed minimal antioxidant activity in the headspace mode. These findings established that headspace TA release was the primary mechanism of antioxidant protection and that PTB-2 provided the most favorable balance of initial burst and sustained release, supporting its selection as the optimal formulation for fruit preservation. The headspace scavenging of PTB-2 (66.85% at 48 h) was within the range reported for rosmarinic acid-crosslinked gelatin films (34.56–87.69%) (Qiao et al., 2024). It should be noted that those values were obtained by direct immersion of films in DPPH solution for 30 min, whereas the present assay measured sustained moisture-mediated headspace transfer over 48 h, a more stringent configuration that reflects actual active packaging conditions.

### 3.6 Antibacterial activity

The antibacterial performance of PTB-2 was evaluated against *S. aureus* and *E. coli* by broth co-culture with OD₆₀₀ monitoring and spread-plate confirmation (Fig. 6A to C). Spread-plate images after 24 h showed markedly reduced colony formation in PTB-2 co-cultures compared with confluent growth in blank controls for both strains. Untreated cultures exhibited exponential proliferation, reaching OD₆₀₀ values of 0.87 ± 0.03 for *S. aureus* and 0.98 ± 0.02 for *E. coli* at 24 h. In contrast, PTB-2 maintained OD₆₀₀ below 0.17 throughout the 24 h period for both strains, with values of 0.07 ± 0.01 and 0.09 ± 0.01 at 24 h, respectively. PB co-cultures remained comparable to the blank (OD₆₀₀ 0.84 ± 0.05 for *S. aureus*; 1.01 ± 0.03 for *E. coli* at 24 h), attributing the inhibition specifically to released TA rather than the PCP matrix. The slight decline in optical density after 18 h suggested that TA exerted a bactericidal rather than merely bacteriostatic effect. The antibacterial mechanism was attributed to the phenolic hydroxyl groups of TA, which can disrupt bacterial membrane integrity, bind to essential proteins, and generate oxidative stress (Xie et al., 2025). These results demonstrated that the released TA from PTB-2 could effectively suppress both Gram-positive and Gram-negative bacteria, reducing the risk of microbial spoilage during fruit storage.

**Fig. 6:**
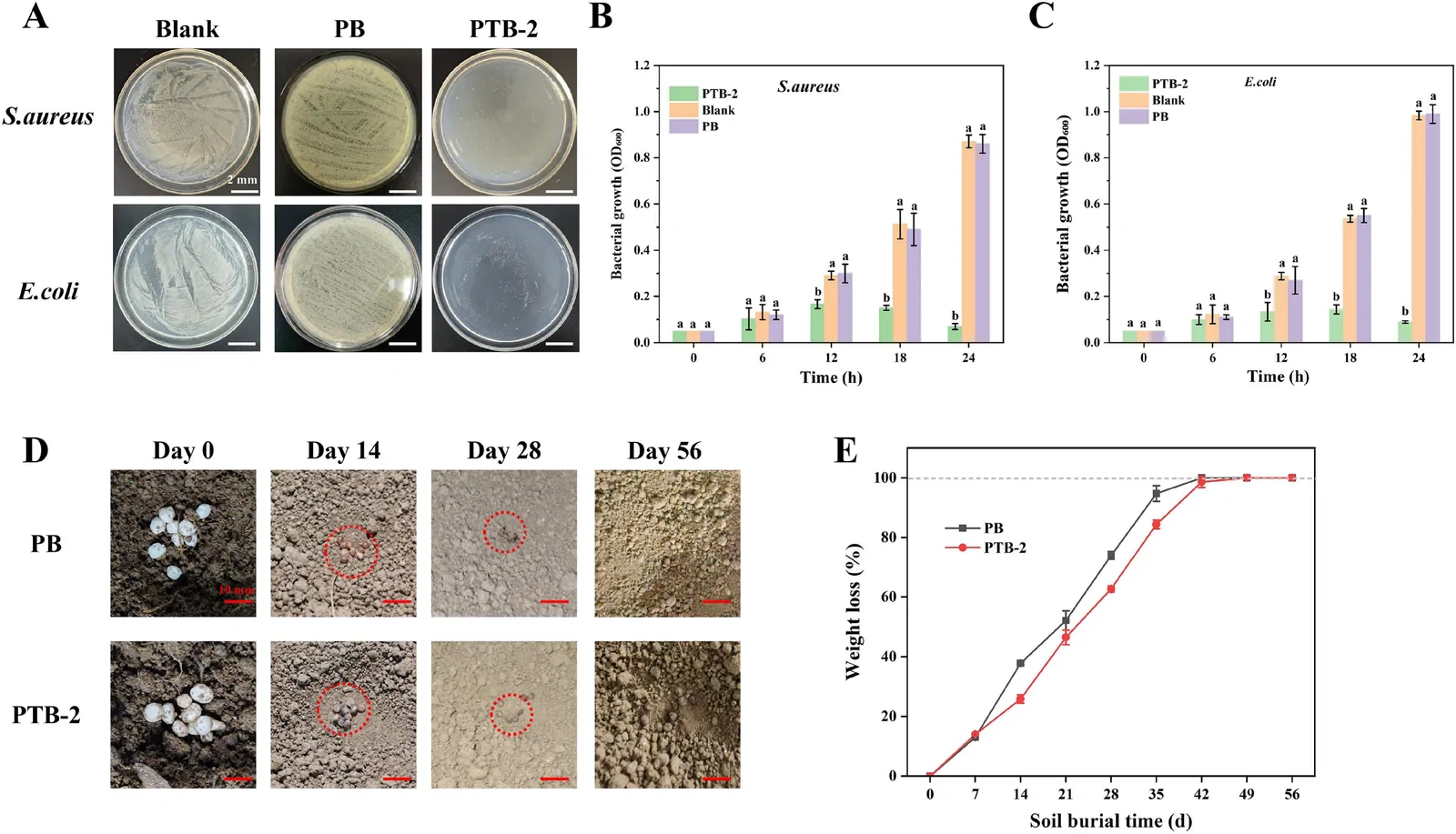
Antibacterial activity and soil burial degradation. (A) Spread-plate images of *S. aureus* and *E. coli*. Scale bar: 2 mm. (B) Bacterial growth of *S. aureus* measured by OD₆₀₀ over 24 h. (C) Bacterial growth of *E. coli* measured by OD₆₀₀ over 24 h. (D) Photographs of PB and PTB-2 during soil burial at days 0, 14, 28, and 56. Scale bar: 10 mm. (E) Weight loss of PB and PTB-2 during 56 days of soil burial. Data are presented as mean ± SD. Different letters indicate significant differences at *p* < 0.05.

### 3.7 Degradation behavior

Freeze-dried beads represent the stable storage state; hydrated beads as used in packages are expected to degrade faster, so the present data give a conservative estimate of environmental persistence. Soil burial tests showed that both PB and PTB-2 were fully biodegradable, but TA crosslinking moderately prolonged the degradation period (Fig. 6D and E). PB exhibited a weight loss of 13.04 ± 0.21% at day 7, rising to 73.92 ± 1.33% by day 28 and reaching complete degradation by day 42. PTB-2 showed a comparable initial loss of 13.97 ± 0.47% at day 7 but degraded more slowly thereafter, recording 62.71 ± 1.02% at day 28 and 84.43 ± 1.48% at day 35 before reaching 100% at day 49. The visual records confirmed that both groups fragmented and progressively disappeared within the soil matrix, with no visible residue remaining at day 56. The delayed degradation of PTB-2 was consistent with its denser hydrogen-bonded network and lower porosity, which restricted water penetration and microbial access to the polymer chains. This moderate degradation rate is advantageous for fruit preservation, as the beads must retain structural integrity and sustained TA release throughout the typical storage and distribution window of two to four weeks while ultimately decomposing without environmental residue. The food-grade ingredients, drop extrusion, and salt leaching are compatible with continuous nozzle scale-up, whereas lyophilization and DMSO currently add energy and solvent-recovery costs; residual DMSO was not detected by ^1^H NMR (Fig. S1), and an all-aqueous dissolution route is targeted in process optimization.

### 3.8 Whole fruit preservation during long-term storage

The preservation efficacy of PTB-2 was evaluated on whole Red Globe grapes and cherry tomatoes over 14 days at 25 °C (Fig. 7), with UVASYS, a commercial sulfur dioxide releasing pad and established industry standard for table grape preservation, included as a positive control.

**Fig. 7:**
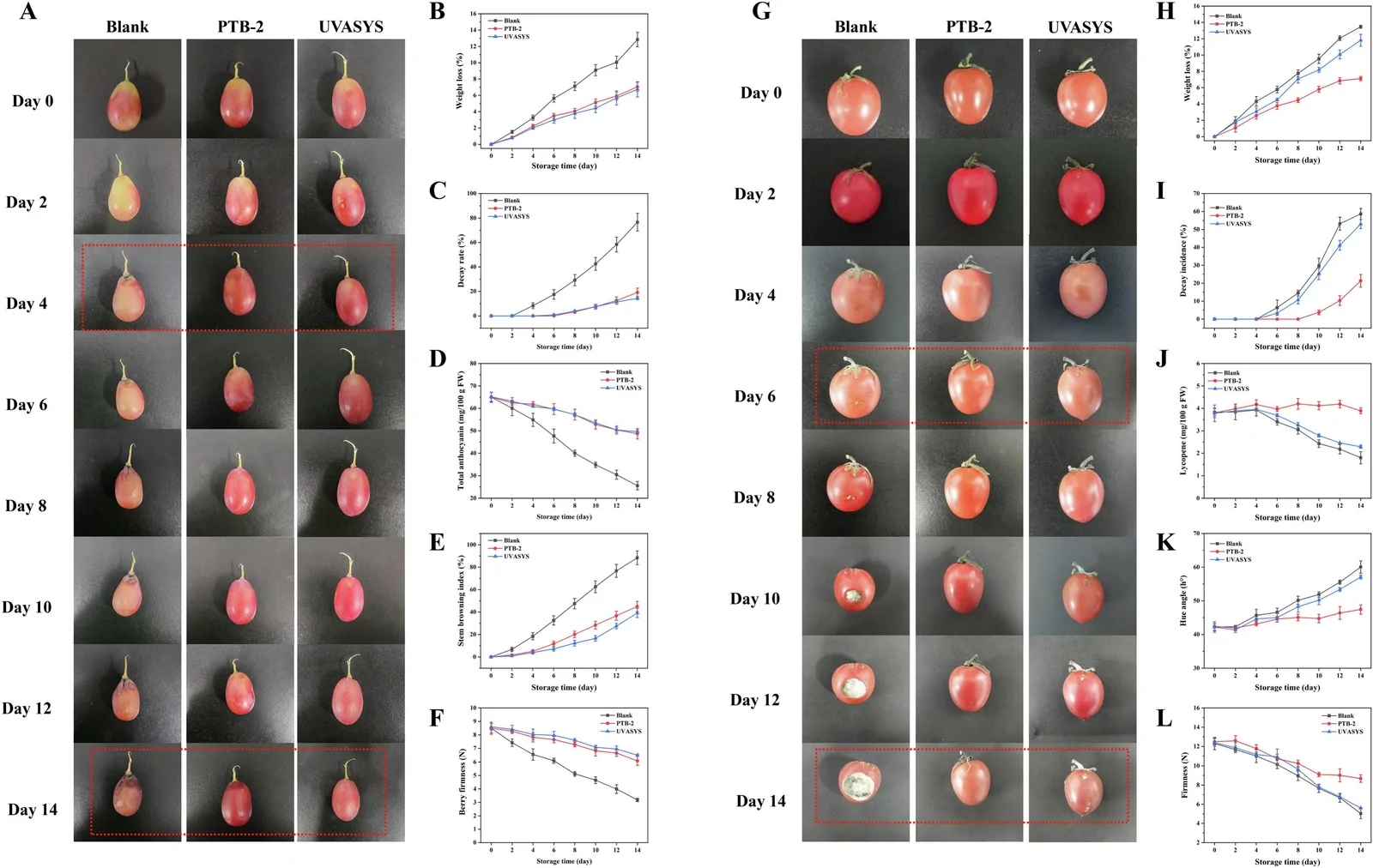
Preservation efficacy of PTB-2 on whole fruit during 14 days at 25 °C storage with UVASYS as a commercial positive control. (A) Visual appearance of Red Globe grapes. (B) Weight loss. (C) Decay incidence. (D) Total anthocyanin content. (E) Stem browning index. (F) Berry firmness. (G) Visual appearance of cherry tomatoes. (H) Weight loss. (I) Decay incidence. (J) Lycopene content. (K) Hue angle. (L) Firmness. Data are presented as mean ± SD (*n* = 3). Different lowercase letters indicate significant differences among treatment groups at each time point at *p* < 0.05. (For interpretation of the references to color in this figure legend, the reader is referred to the web version of this article.)

For Red Globe grapes, both PTB-2 and UVASYS substantially outperformed the blank across all quality indices. Weight loss in the blank reached 12.84 ± 0.88% by day 14, whereas PTB-2 and UVASYS limited weight loss to 7.05 ± 0.55% and 6.74 ± 0.93%, reductions of 45.1% and 47.5%, respectively (Fig. 7B). Decay incidence in the blank escalated from day 4 to 76.67 ± 7.24% by day 14. PTB-2 delayed decay onset to day 8 and held the terminal decay incidence at 19.17 ± 3.52%, while UVASYS delayed onset to day 6 and achieved 14.24 ± 0.99% (Fig. 7C). Total anthocyanin content followed a comparable pattern, decreasing from 65.05 to 25.55 ± 1.85 mg per 100 g in the blank but remaining at 48.69 ± 2.33 and 49.65 ± 0.82 mg per 100 g under PTB-2 and UVASYS (Fig. 7D). Stem browning reached 88.33 ± 6.15% in the blank compared with 45.07 ± 4.56% under PTB-2 and 39.11 ± 4.05% under UVASYS (Fig. 7E). Berry firmness declined from 8.50 to 3.18 ± 0.12 N in the blank, while PTB-2 and UVASYS retained 6.08 ± 0.33 and 6.49 ± 0.07 N, corresponding to 1.91-fold and 2.04-fold improvements (Fig. 7F). The performance of PTB-2 was statistically comparable to UVASYS on grapes, with no significant difference at any time point and no significant difference in anthocyanin retention throughout storage. This equivalence is notable because UVASYS relies on synthetic sulfite chemistry that has dominated table grape preservation for decades, whereas PTB-2 achieves the same outcome through a natural polyphenol without relying on sulfite chemistry. The 75% reduction in grape decay achieved by PTB-2 compared favorably with the 52.3% reduction reported for iturin A/chitosan microcapsules over 16 days (Li et al., 2024), indicating competitive preservation efficacy relative to established bio-based coating systems.

For cherry tomatoes, PTB-2 demonstrated a clear and substantial advantage over both the blank and UVASYS. Weight loss in the blank and UVASYS groups reached 13.47 ± 0.18% and 11.81 ± 0.73% by day 14, while PTB-2 reduced weight loss to 7.11 ± 0.26%, which was 47.2% lower than the blank and 39.8% lower than UVASYS (Fig. 7H). The 47.2% weight loss reduction in cherry tomatoes exceeded the 29.77% reduction achieved by rosmarinic acid-crosslinked gelatin films (Qiao et al., 2024). Decay incidence provided the most striking contrast. The blank reached 58.63 ± 3.06% decay by day 14, and UVASYS provided only marginal protection at 53.08 ± 2.54%, a mere 9.5% improvement over the blank. PTB-2 delayed decay onset to day 10 and reduced terminal decay to 21.33 ± 3.55%, representing 63.6% and 59.8% reductions relative to the blank and UVASYS, respectively (Fig. 7I). Lycopene content in the blank decreased from 3.83 to 1.80 ± 0.27 mg per 100 g, and UVASYS showed limited benefit at 2.29 ± 0.07 mg per 100 g. PTB-2 preserved lycopene at 3.89 ± 0.13 mg per 100 g, effectively stabilizing the pigment at its initial level as the fruit underwent normal ripening (Fig. 7J). Hue angle remained stable at 47.43 ± 1.39° under PTB-2 but increased to 60.05 ± 1.81° in the blank and 57.09 ± 0.57° under UVASYS, reflecting progressive surface darkening and senescence in the latter groups (Fig. 7K). Firmness declined from 12.30 to 5.04 ± 0.56 N in the blank and 5.61 ± 0.05 N under UVASYS, while PTB-2 maintained firmness at 8.68 ± 0.39 N, 1.72-fold and 1.55-fold higher than the two controls (Fig. 7L). Visual records confirmed that blank and UVASYS-treated tomatoes developed extensive fungal colonies by day 12, whereas PTB-2-treated fruit retained an intact and glossy appearance through day 14. All tomatoes continued normal red color development during the first 2 days, consistent with climacteric ripening and showing that PTB-2 did not arrest ripening. Direct residue analysis detected only trace TA on intact fruit surfaces (2.07–6.26 μg TA eq./g FW over 7–14 days), whereas TA remained below the quantification limit in the flesh and in untreated or UVASYS controls throughout storage (Table S1), indicating non-penetrating, residue-minimized exposure. Because PTB-2 and UVASYS deliver different agents by different routes, the comparison addresses overall preservation performance rather than equimolar antimicrobial exposure.

The divergent performance of UVASYS between the two fruit types arises from the mechanism of sulfur dioxide action. Sulfur dioxide exerts antifungal activity through sulfite diffusion across fruit skin, which is effective on the thin permeable cuticle of grapes but substantially impeded by the thick waxy surface and higher pH of tomato fruit (Daniel-Swartland et al., 2024). SO₂ is highly active against *Botrytis cinerea*, the dominant grape pathogen, whereas the principal tomato spoilage fungi (*Rhizopus, Alternaria* spp.) are far less SO₂-sensitive, and tomato tissue is additionally prone to SO₂ bleaching injury. PTB-2 functions through headspace release of tannic acid, whose antioxidant and membrane-disrupting activities operate independently of fruit surface morphology (Xie et al., 2025). The perforation density was chosen in preliminary trials to hold 85–95% RH without anaerobic condensation; the recovery of TA on fruit surfaces under this identical perforated configuration (Table S1) confirms that sufficient active material is retained despite ventilation. The comparable efficacy on grapes validates that a natural polyphenol system can match a synthetic industry standard without SO₂ release, while the superior performance on tomatoes reveals an application range that sulfur dioxide technology cannot achieve. These findings establish PTB-2 as a versatile, residue-minimizing preservation platform suitable for diverse fruit commodities.

### 3.9 Food-contact safety assessment

Food-contact safety was assessed by 28-day repeated oral administration at 2 g/kg body weight per day (Fig. 8). Both groups exhibited steady and comparable weight gain throughout the feeding period. Body weight increased from 22.34 ± 0.85 to 32.81 ± 0.56 g in the control and from 23.13 ± 1.09 to 34.14 ± 0.49 g in the PTB-2 group, with no significant difference at any time point (Fig. 8B). Organ indices for heart, liver, spleen, lung, and kidney were nearly identical between groups, with liver values of 4.76 ± 0.29% for the control and 4.81 ± 0.29% for PTB-2, and kidney values of 1.29 ± 0.09% and 1.26 ± 0.09%, respectively (Fig. 8C). H&E staining revealed normal tissue architecture in liver, kidney, and spleen of PTB-2-treated mice, with no inflammatory infiltration, necrosis, or structural abnormalities compared with the control (Fig. 8A).

**Fig. 8:**
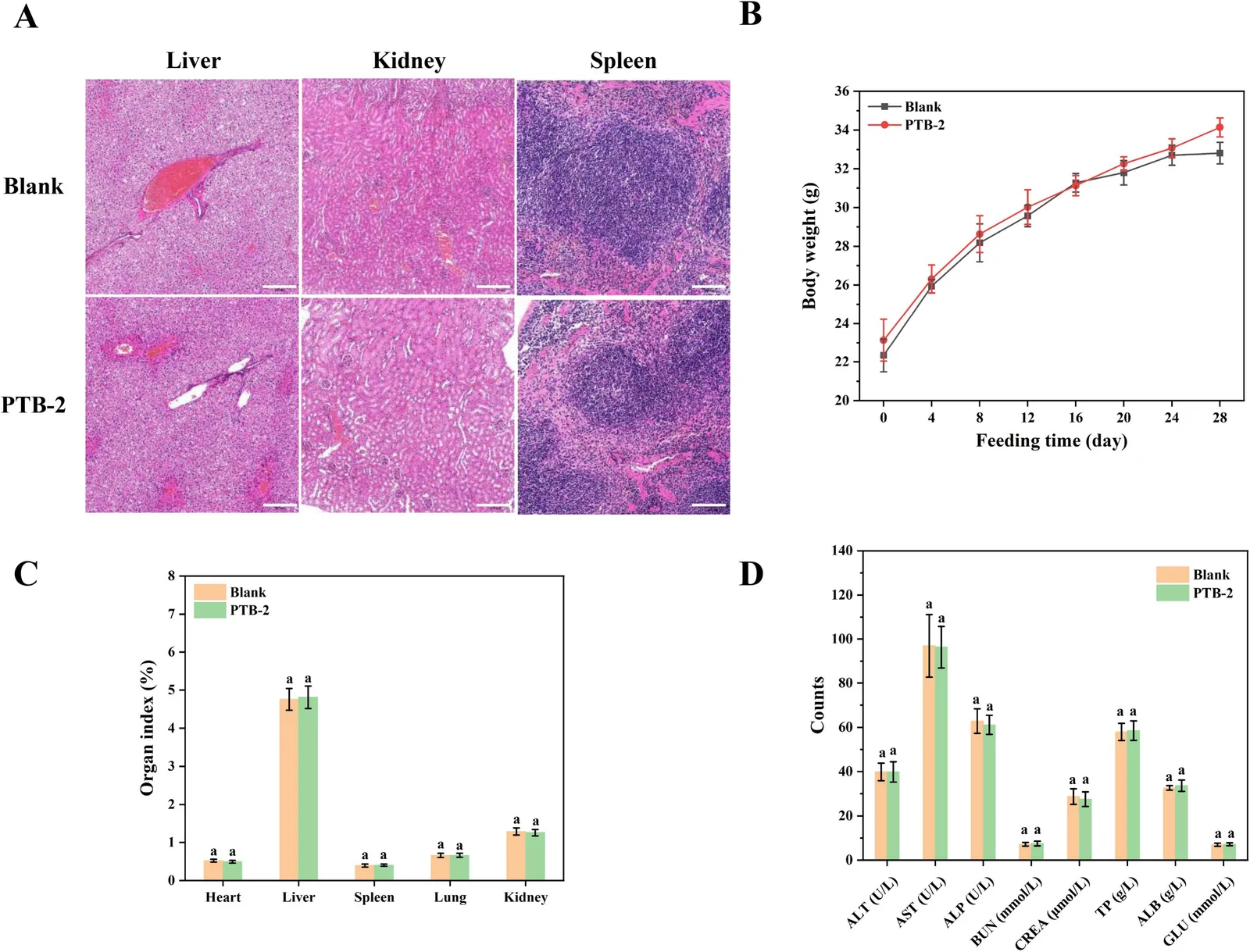
In vivo biosafety evaluation of PTB-2 after 28 days of oral administration. (A) H&E staining of liver, kidney, and spleen tissues. Scale bar: 100 μm. (B) Body weight curves over 28 days. (C) Organ indices of heart, liver, spleen, lung, and kidney. (D) Serum biochemical parameters including ALT, AST, ALP, BUN, CREA, TP, ALB, and GLU. Data are presented as mean ± SD. No significant differences were observed between groups at *p* < 0.05.

Serum biochemistry confirmed the absence of hepatic or renal toxicity. Liver function markers ALT and AST were 39.84 ± 3.98 and 96.97 ± 14.20 U/L in the control versus 39.88 ± 4.60 and 96.36 ± 9.48 U/L in the PTB-2 group. Renal function markers BUN and CREA showed similar equivalence at 7.18 ± 0.83 mmol/L and 28.73 ± 3.54 μmol/L for the control compared with 7.52 ± 1.09 mmol/L and 27.56 ± 3.30 μmol/L for PTB-2. Total protein, albumin, and glucose levels also fell within normal ranges and did not differ significantly between groups (Fig. 8D). These findings were consistent with recent biosafety studies of polysaccharide-polyphenol hydrogels, where starch-catechol implants showed no histopathological abnormalities in major organs after 28 days (An et al., 2025), and oral administration of nanozyme-loaded calcium alginate hydrogel microspheres produced normal body weight gain, blood chemistry, and organ histology in mice (Fan et al., 2026). The absence of adverse effects across body weight, organ indices, histology, and serum chemistry demonstrated an absence of detectable acute oral or systemic toxicity under this worst-case exposure, supporting the intended non-contact packaging application, while comprehensive food-contact conclusions would require standardized migration testing.

## 4 Conclusion

In this study, porous *Poria cocos* polysaccharide/tannic acid hydrogel beads with tunable pore architecture were successfully fabricated through salt porogen leaching and gradient annealing. The medium-porosity PTB-2 combined uniform interconnected pores with high moisture sorption and achieved 97.4% cumulative tannic acid release governed by anomalous transport, delivering sustained headspace antioxidant activity over 48 h. The pore-dependent release mechanism, quantified by Korsmeyer-Peppas analysis, enabled rational optimization of the matrix without altering composition. Over 14 days of room-temperature storage, PTB-2 reduced grape decay to 19.2%, matching the performance of the commercial UVASYS sulfur dioxide pad without the need for SO₂-generating chemistry, while on cherry tomatoes it reduced decay to 21.3%, substantially outperforming UVASYS at 53.1%. This broad-spectrum efficacy across a non-climacteric berry and a climacteric fruit distinguished the headspace polyphenol delivery mechanism from surface-dependent synthetic preservatives. The beads were fully biodegradable within 49 days, and 28-day oral administration in mice revealed no systemic toxicity. These findings demonstrate that pore architecture is a tunable structural lever governing polyphenol mass transfer from a bio-based polysaccharide matrix, and that pore-engineered PCP/TA beads can serve as a natural and biodegradable active packaging platform, supported by trace-only non-penetrating residues and no detectable acute oral toxicity under the tested conditions, that can complement or replace synthetic preservatives across diverse fruit commodities.

## Declaration of generative AI and AI-assisted technologies in the manuscript preparation process

During the preparation of this work, the authors used Coze (an AI assistant developed by ByteDance) for English language polishing, academic phrasing revision, manuscript text refinement, and generation of schematic illustrations. After using these tools, the authors reviewed and edited the content thoroughly and take full responsibility for the content of the published article.

## CRediT authorship contribution statement

**Xichen Qiao:** Writing – original draft, Visualization, Software, Methodology, Investigation, Formal analysis. **Jinwang Zou:** Writing – original draft, Resources, Methodology, Investigation, Formal analysis. **You Zhang:** Methodology, Data curation. **Chang Zhou:** Software, Data curation. **Ruyan Qian:** Methodology, Data curation. **Xizun Tan:** Methodology, Data curation. **Shipeng Wu:** Investigation, Formal analysis. **Changchun Li:** Supervision, Funding acquisition. **Yao Wu:** Supervision, Software. **Hairong Xiong:** Supervision, Methodology, Funding acquisition. **Zongbao Zhou:** Writing – review & editing, Supervision, Project administration, Funding acquisition, Conceptualization.

## Ethics declaration

### Animal subject

This study was conducted in accordance with the ARRIVE (Animal Research: Reporting of In Vivo Experiments) guidelines.

This study was approved by the Ethics Committee of Hubei Engineering University (Approval No. A2026010).

## Declaration of competing interest

The authors declare that they have no known competing financial interests or personal relationships that could have appeared to influence the work reported in this paper.

## Data Availability

Data will be made available on request.

## Appendix A Supplementary data

Supplementary material Additional methods (Method S1–S3), Figure S1–S3, and Table S1 are provided as Supplementary Material.
