# Supplementary material for: Pore architecture governs tannic acid release from porous Poria cocos polysaccharide beads for headspace antioxidant preservation of fruit
Source: Food Chem X. 2026 Oct 22;40:104670. doi: 10.1016/j.fochx.2026.104670 (PMC13718855; doi:10.1016/j.fochx.2026.104670)
Supplement: Supplementary file 1 [file mmc1.docx]

**Pore architecture governs tannic acid release from porous *Poria cocos* polysaccharide beads for headspace antioxidant preservation of fruit**

**Xichen Qiao ^1, a^, Jinwang Zou ^1, a^, You Zhang ^a^, Chang Zhou ^a^, Ruyan Qian ^a^, Xizun Tan ^a^, Shipeng Wu ^b^, Changchun Li** **^a^, Yao Wu** **^a^, Hairong Xiong ^a^, and Zongbao Zhou ^a,^***

^a^ *Hubei Key Laboratory of Resource Utilization and Quality Control of Characteristic Crops, College of Life Science and Technology, Hubei Engineering University, Xiaogan 432000, China*

^b^ *Department of Stomatology, Shanghai Eighth People’s Hospital, Jiangsu University, Shanghai 200235, China*

*Corresponding Author: *Hubei Key Laboratory of Resource Utilization and Quality Control of Characteristic Crops, Hubei Engineering University, Xiaogan 432000, China*.

*E-mail addresses*: [zzb1991@hbeu.edu.cn](mailto:zzb1991@hbeu.edu.cn) (Z. Zhou).

1. **Method**

**S1. Residual DMSO analysis**

Residual DMSO in the beads was examined by ¹H NMR. Lyophilized PTB-2 beads (0.30 g) were extracted with 1.0 mL D₂O at 25 °C and centrifuged, and the supernatant was analyzed with D₂O lock. A DMSO solution in D₂O producing the characteristic six-proton singlet at δ ~2.6 ppm served as the positive reference, and D₂O alone served as the blank. No DMSO signal was detected in the PTB-2 extract, indicating that residual DMSO was effectively removed during washing (Fig. S1).

**S2. Determination of TA loading content and encapsulation efficiency**

Lyophilized beads were ground, and approximately 30 mg powder was extracted three times with 5 mL of 50% (v/v) methanol under 30 min ultrasonication followed by 1 h incubation at 50 °C. After centrifugation at 10000 rpm for 10 min, supernatants were combined and TA was quantified by the Folin-Ciocalteu method at 760 nm using a TA calibration curve, with PB beads processed identically as the matrix blank. Loading content (LC) and encapsulation efficiency (EE) were calculated as:

$$\begin{aligned} \text{LC}\text{ (\%) = }\frac{\text{W}_{\text{TA}}}{\text{W}_{\text{bead}}}\text{×100}\# \end{aligned}$$

$$\begin{aligned} \text{EE}\text{ (\%) = }\frac{\text{W}_{\text{TA}}}{\text{W}_{\text{feed}}}\text{×100}\# \end{aligned}$$

where W_TA_, W_bead_, and W_feed_ are the extracted TA mass, dry bead mass, and initially fed TA mass. Three independently fabricated batches were measured.

**S3. Determination of TA residues on fruit surfaces and in flesh**

At days 7 and 14, three independent packages per treatment (Control, UVASYS, and PTB-2; 200 g grape or 150 g cherry tomato per package) were sampled. For surface residues, intact fruit were rinsed with a fixed volume of 70% (v/v) methanol containing 0.1% formic acid (20 mL per 100 g fruit) and gently shaken for 5 min; the rinse was repeated once, and the combined rinses were concentrated under reduced pressure and reconstituted to a fixed volume. For flesh residues, the peel was removed, the flesh was homogenized and extracted with the same solvent, and the extract was clarified by centrifugation at 10000 rpm for 10 min. TA was quantified by the Folin-Ciocalteu method at 760 nm against a TA calibration curve. Rinses and flesh extracts of untreated fruit, processed identically on the same day, served as matrix-matched blanks and were subtracted to exclude endogenous fruit phenolics. Limits of detection and quantification were determined at signal-to-noise ratios of 3 and 10, respectively. Method accuracy was evaluated by spiking untreated-fruit rinses with 5, 20, and 50 µg TA (n = 3), with recoveries of 85.9–100.7%. Values below the LOQ were reported as ND.

2. **Result**

**Figure S1**. ¹H NMR spectra, from top to bottom: D₂O blank, D₂O extract of PTB-2 beads, and DMSO reference in D₂O. The DMSO reference gives the characteristic six-proton singlet at δ ~2.6 ppm; the signal at δ 4.7 ppm is HDO. No DMSO signal is observed in the PTB-2 extract.

**Figure S2**. Actual TA loading content (LC, A) and encapsulation efficiency (EE, B) of PTB-1, PTB-2, and PTB-3 (mean ± SD, n = 3 independent batches). Different letters indicate significant differences (p < 0.05).

**Table S1**. Tannic acid residues on the surface and in the flesh of Red Globe grape and cherry tomato after 7 and 14 days of storage at 25 °C (µg TA eq./g FW, mean ± SD, n = 3 independent packages).

| **Fruit** | **Tissue** | **Day** | **Control** | **UVASYS** | **PTB-2** |
| --- | --- | --- | --- | --- | --- |
| Red Globe grape | Surface | 7 | ND | ND | 2.42 ± 0.52 |
| Red Globe grape | Surface | 14 | ND | ND | 6.26 ± 0.93 |
| Red Globe grape | Flesh | 7 | ND | ND | ND |
| Red Globe grape | Flesh | 14 | ND | ND | ND |
| Cherry tomato | Surface | 7 | ND | ND | 2.07 ± 0.84 |
| Cherry tomato | Surface | 14 | ND | ND | 4.35 ± 1.79 |
| Cherry tomato | Flesh | 7 | ND | ND | ND |
| Cherry tomato | Flesh | 14 | ND | ND | ND |

Note: ND, not detected (below the limit of quantification, LOQ = 2.0 µg/g FW). Values were background-corrected against rinses or extracts of untreated fruit processed in parallel as matrix-matched blanks. TA was quantified by the Folin-Ciocalteu method at 760 nm using a TA calibration curve.

**Figure S3.** Handling robustness of hydrated PTB-2 beads. (i) Bead held between tweezers, (ii) manually compressed, and (iii) released. The bead recovered its shape without rupture or fragmentation after compression.
